# Correction: A SYK/SHC1 pathway regulates the amount of CFTR in the plasma membrane

**DOI:** 10.1007/s00018-024-05360-7

**Published:** 2024-08-01

**Authors:** Cláudia Almeida Loureiro, Francisco R. Pinto, Patrícia Barros, Paulo Matos, Peter Jordan

**Affiliations:** 1 Department of Human Genetics, National Health Institute ‘Dr. Ricardo Jorge’, Avenida Padre Cruz, Lisbon 1649-016 Portugal; 2 BioISI-Biosystems and Integrative Sciences Institute, Faculty of Sciences, University of Lisbon, Lisbon, Portugal; 3 Department of Chemistry and Biochemistry, Faculty of Sciences, University of Lisbon, Lisbon, Portugal

**Correction to: Cellular and Molecular Life Sciences (2020) 77:4997–5015**

10.1007/s00018-020-03448-4

The authors were notified by the editors of reader concerns regarding a few Western blot panels in some of the figures of the published article.

All original Western blot films were shared with the editors, and after conducting a thorough review in collaboration with the editors, issues requiring correction were identified. During the assembly of multi-layer Fig. 5b the panels representing the input lysates were inadvertently duplicated from those used in Fig. 6a. In Figs. 1, 2a and 4, and 5a image quality problems or incongruences between the figure panels and their legends were identified.

Based on the original data, the corrected versions of Figs. 1, 2a and d, 4, 5a and b and 6 are shown below, including the revision of some corresponding legends.

The authors apologize for the mistakes and any inconvenience they may have caused, but note that the issues do not in any way affect the scientific conclusions drawn from the experimental data.

The original article has been corrected.

**Fig. 1:**
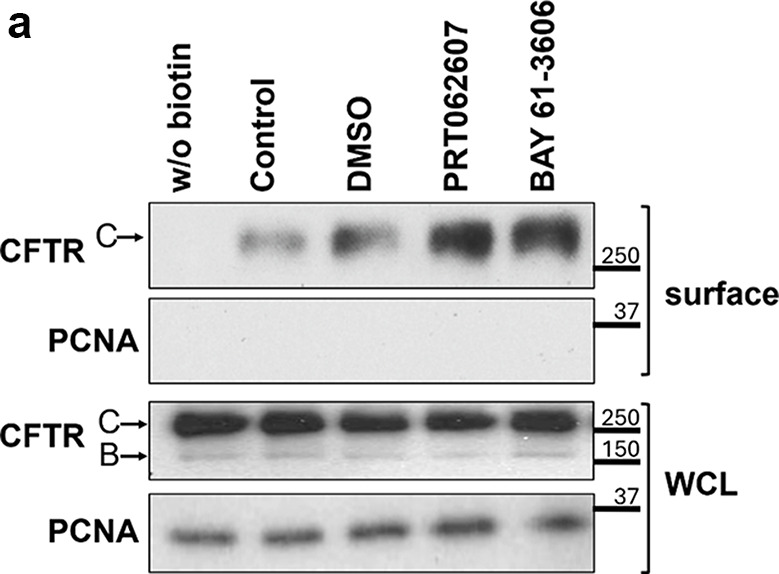
.

**Fig. 2:**
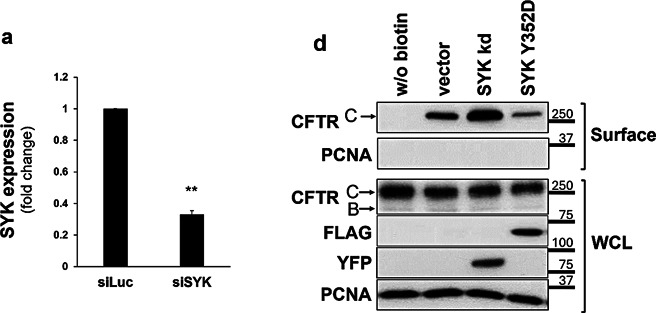
**a** Assessment of the efficiency of siRNA-mediated knockdown of SYK. CFBE wt-CFTR cells were transfected as described under “Cell culture and transfections” and depletion of endogenous SYK protein levels analyzed by Western blot (representative image shown in **b**). Data quantification by Student’s *t* test (*n* = 6) is graphically displayed and shown as fold-change relative to siLUC (control). Note the successful downregulation of SYK expression by approximately 70%

**Fig. 4:**
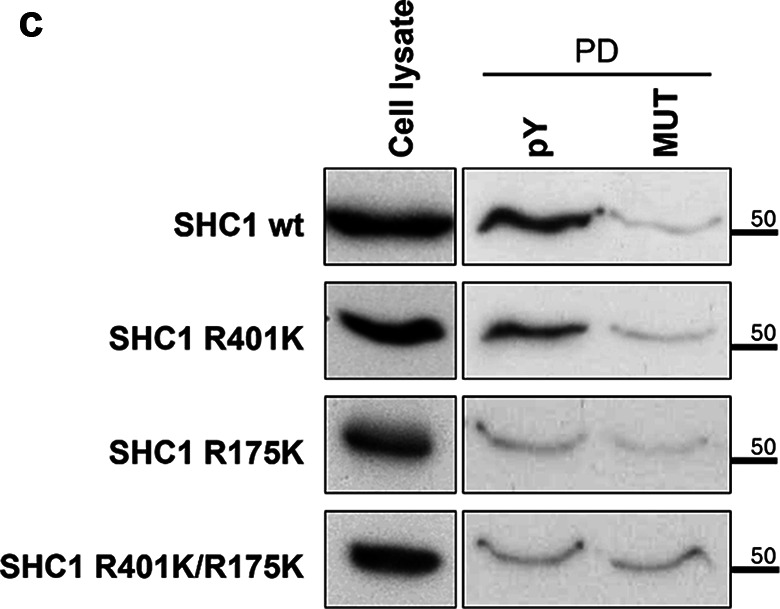
**c** The PTB domain of SHC1 is required for association with CFTR phosphotyrosine domain. WB analysis following the PPD assay with lysates from CFBE wt-CFTR cells previously transfected with the indicated Myc-tagged SHC1 constructs (wt or mutant). Note that the point mutation R175K in the SHC1 PTB domain abolishes binding to the phosphorylated peptide. **d** SHC1 forms a protein complex with CFTR depending on SYK protein kinase activity. CFBE mCherry-Flag-wt-CFTR cells were co-transfected with Myc-SHC1 and either constitutively active SYK Y352D or SYK kd mutants. The fraction of PM-localized CFTR was immunoprecipitated with anti-Flag antibody (IP) and the amount of SHC1 co-precipitating with CFTR analyzed by WB. Note that the presence of active SYK strongly increased the co-precipitation of SHC1 with the mature, fully glycosylated CFTR-band C. Input- whole cell lysates; IP- immunoprecipitated fractions; Ab- antibody; EV – empty vector. All data shown are representative for at least three independent experiments

**Fig. 5:**
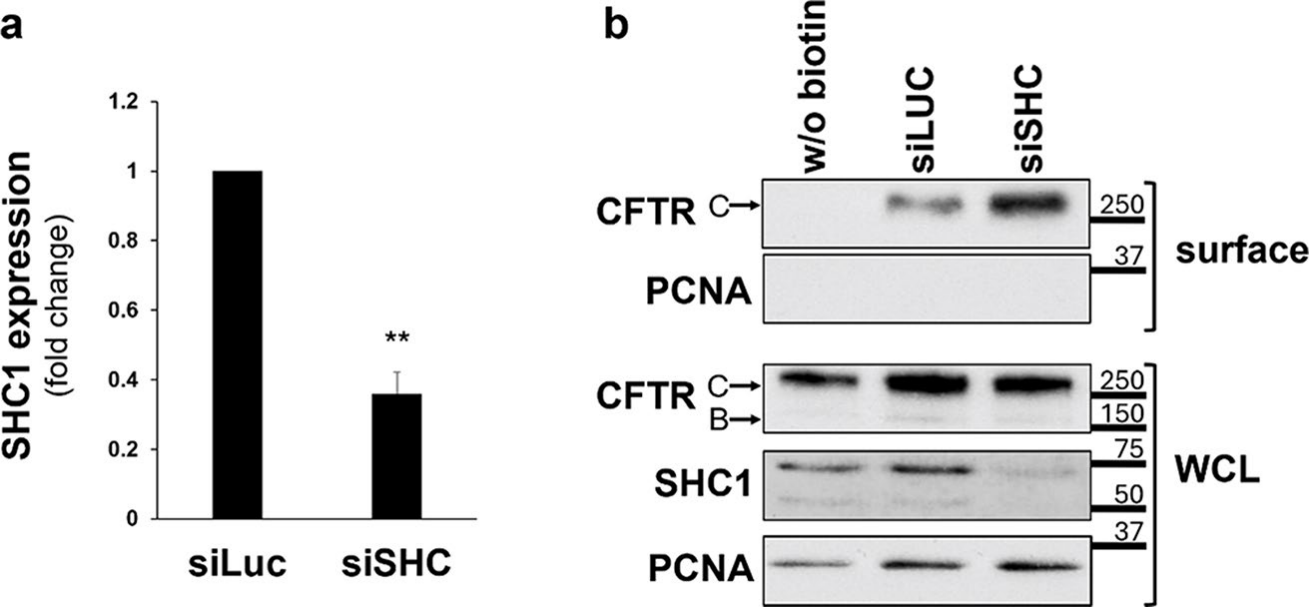
**a** Assessment of the efficiency of siRNA-mediated knockdown of SHC1. CFBE wt-CFTR cells were transfected as described under “Cell culture and transfections” and depletion of endogenous SHC1 protein levels analyzed by Western blot (representative image shown in **b**). Data quantification (*n* = 6), analyzed by Student’s t test, is graphically displayed and shown as fold-change relative to siLUC (control). Note the successful downregulation of SHC1 expression by approximately 61%

**Fig. 6:**
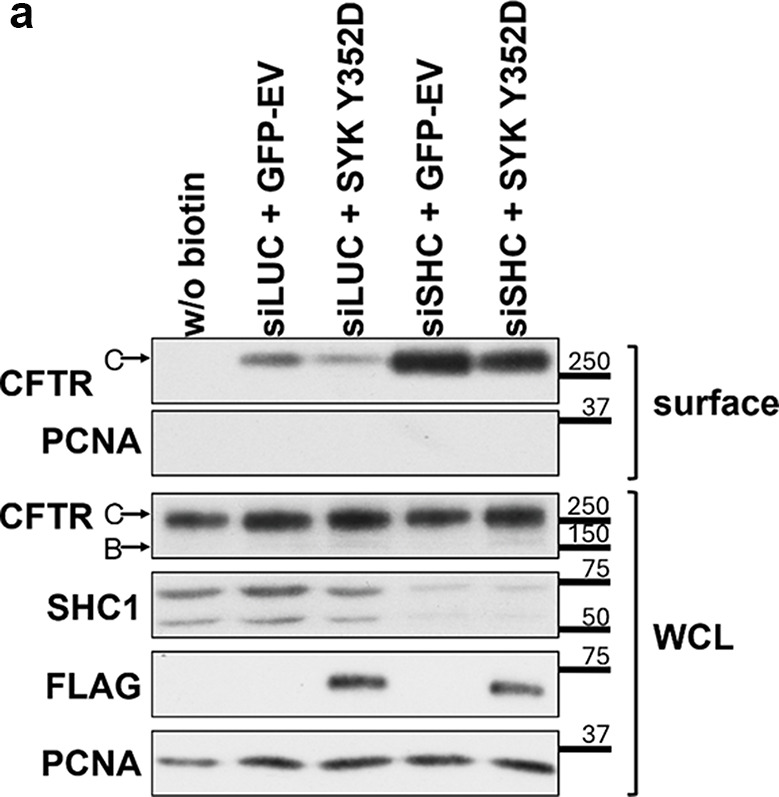
.

